# Synthesis Strategy Toward Minimizing Adventitious Oxygen Contents in the Mechanochemically Made Semiconductor Kesterite Cu_2_ZnSnS_4_ Nanopowders

**DOI:** 10.3390/ma17246091

**Published:** 2024-12-13

**Authors:** Katarzyna Kapusta, Zbigniew Olejniczak, Jerzy F. Janik

**Affiliations:** 1 Faculty of Energy and Fuels, AGH University, al. Mickiewicza 30, 30 059 Kraków, Poland; kkapusta@agh.edu.pl; 2 Institute of Nuclear Physics, Polish Academy of Sciences, ul. Radzikowskiego 152, 31 342 Krakow, Poland; zbigniew.olejniczak@ifj.edu.pl

**Keywords:** kesterite semiconductor, photovoltaics, optimized mechanochemical synthesis, residual oxygen content, oxygen footprint

## Abstract

A multipronged approach to the refined mechanochemical synthesis of the semiconductor kesterite Cu_2_ZnSnS_4_ with minimal quantities of adventitious oxygen as well as to optimizing handling procedures from that angle is described. Three precursor systems are used to provide a pool of freshly made cubic prekesterite nanopowders with no semiconductor properties and the thermally annealed at 500 °C tetragonal kesterite nanopowders of the semiconductor. Based on the previously reported high propensity of such nanopowders to long-term deteriorating oxidation in ambient air, suitable modifications of all crucial synthesis steps are implemented, which are directed toward excluding or limiting the materials’ exposure to air. The nanopowders are comprehensively characterized by powder XRD, FT-IR/Raman/UV-Vis spectroscopies, solid-state ^65^Cu/^119^Sn MAS NMR, TGA/DTA-QMS analysis, SEM, BET/BJH specific surface area, and helium density determinations, and, significantly, are directly analyzed for oxygen and hydrogen contents. The important finding is that following the anaerobic procedures and realistically minimizing the materials’ exposure to air in certain manipulation steps results in the preparation of better oxidation-resistant nanopowders with a dramatic relative decrease in their oxygen content than previously reported. The adherence to the strict synthesis conditions that limit contact of the no-oxygen-containing kesterite nanopowders with ambient air is emphasized.

## 1. Introduction

The well-founded potential of the quaternary sulfide Cu_2_ZnSnS_4_ semiconductor, customarily called kesterite, for the next generation of efficient photovoltaic cells and its increasing prospects for thermoelectric applications have shown interesting developments in the area of synthesis and applications while stressing also the compound’s solid environmental advantages [1,2,3,4,5]. The compound is a complex sulfide with characteristics of structural disorder and stoichiometry variations [6,7,8]. These features, in turn, impact its chemical stability properties, including, for instance, doping, the formation of secondary phases, and interface reactivity, especially in the material’s forms of high-surface-area nanopowders and/or prototype thin layers [9,10].

One of the often-overlooked kesterite properties is its susceptibility to deteriorating oxidation under ambient humid air conditions. This problem can become significant both during various stages of the sulfide synthesis and in the subsequent manipulation, characterization, storage, cell manufacturing, and application steps. Despite this, it has not received due attention in suitably focused research. Regarding the aspects remotely related to oxidation, there are a few theoretical studies indicating the possibility of oxygen centers to be incorporated in the kesterite crystal lattice by replacing sulfur atoms in the entire composition range, which eventually impacts the compound’s crucial semiconductor properties [11,12]. In other studies that concentrated on various prototype PV cell structures, including the ideas of converting oxygen-bearing precursor films by sulfurization toward kesterite [13], O for S/Se atoms’ replacement in the crystal lattice by annealing the selenized kesterite films in air [14], or thermally enhanced annealing in air of certain kesterite-based PV cells [15], it was reported that such intentional oxidation of kesterite layers at elevated temperatures might influence multidirectionally their optoelectronic properties, stability, and efficiency.

Given the uncharted area of kesterite susceptibility to oxidation in ambient air, we recently embarked on investigations of the oxidation-related deteriorating phenomena associated with the kesterite nanopowder’s exposure to air. Initially, as is customary, we were following the conventional synthesis route that assumed kesterite chemical stability/inertness under such conditions while concentrating on the material’s oxygen footprint description [16,17,18]. For the nanopowder preparation, we used the convenient mechanochemical synthesis method with high-energy ball milling utilizing three precursor systems [19,20,21]. Therein, we found in all cases that the raw product identified after milling, tentatively called prekesterite, is a cubic polytype with defunct semiconductor properties as measured by the UV-Vis methodology. Upon annealing under argon in the 500–550° range, this polytype undergoes phase conversion to the tetragonal variety of the semiconductor, called kesterite, with the expected energy bandgap in the range 1.3–1.5 eV. The results of the kesterite oxidation study for both polytypes unequivocally confirmed the high susceptibility of the nanopowders to react in the ambient air toward copper and zinc hydrated sulfates, whereas the tin component was found in the form of hydrated tin(IV) oxide. The significant oxidation progress was noted already after one month of exposure in all preparation routes with ca. 22–28 wt% analyzed oxygen contents (O-contents) that after six months reached the 32–36 wt% levels. This has to be compared with the initial 4–6 wt% O-contents in the raw cubic polytypes and the 0.6–1.6 wt% O-contents in the annealed tetragonal polytypes, which confirm a higher reactivity of the raw vs. annealed nanopowders. Interestingly, the remaining unreacted tetragonal kesterite still showed by UV-Vis spectroscopy the typical semiconductor properties but with up to a 0.2 eV shift of the energy bandgap to lower values.

Herein, we re-investigated the mechanochemically assisted preparation of both kesterite polytypes by implementing the suitable process modifications in all three precursor routes, which were designed to eliminate or reduce the deteriorating impact of air on the nanopowders. The crucial changes included the application of anaerobic conditions and storage under argon whenever possible and the limited material exposure times to air in some excusable stages to yield remarkable results.

## 2. Experimental

### 2.1. Air Access-Restricted Mechanochemical Synthesis of Kesterite Nanopowders

The same three precursor systems are applied in the two-step mechanochemical synthesis method (“wet” high-energy ball milling in xylene, planetary mill Pulverisette 7, Fritsch, Idar-Oberstein, Germany) as described in our previous kesterite oxidation study reports [16,17,18]. They are labeled as (i) CE (constituent elements), i.e., copper Cu, zinc Zn, tin Sn, and sulfur S with 2 at% excess of S milled for 16 h at 1000 rpm [19]; (ii) MS (metal sulfides), i.e., copper(I) sulfide Cu_2_S, zinc(II) sulfide ZnS, tin(II) sulfide SnS, and sulfur S with 2 at% excess of S milled for 20 h at 900 rpm [20]; and (iii) CA (copper alloys), i.e., first, the in situ copper alloys are prepared by high-energy ball milling of the metal powders {2Cu + Zn + Sn} for 10 h at 900 rpm, which are next milled with an added 2 at% excess of sulfur vs. stoichiometry for 4 h at 900 rpm [21]. Specifically, in contrast to the standard procedures, we follow this procedure (approximate times of material exposure to air are shown in parentheses):

- The newly acquired commercial batches of the precursors are opened in an argon-filled glove box and weighed there (totaling ca. 7 g) to be placed next in the 20 mL grinding bowl of the planetary mill together with a set of 80 tungsten carbide WC of 5 mm in diameter balls;
- After taking out of the glove box, the bowl is briefly opened to air (<1 min), some 6–7 mL of xylene is quickly added, and the closed bowl is placed in the grinding chamber;
- After milling, the bowl is opened to air and the content (product slush containing the balls) is promptly transferred to an argon-filled Schlenk flask (<2 min);
- The flask’s content is evacuated to dryness on a vacuum line for 0.5 h to remove the xylene and back-filled with argon;
- The flask is then transferred to a glove box to separate under argon the dry powdery solid product from the grinding balls;
- All powders are stored in the glove box throughout the manipulation and characterization steps, whereas samples for characterization are assembled in tightly closed vials under argon;
- Approx. 2 g charges of the cubic prekesterite nanopowders are prepared in the glove box for the thermal treatment under argon toward tetragonal kesterite, which are then transferred in a ceramic boat in air (<1 min) to an argon-flushed quartz tube of the furnace. After the annealing at 500 °C for 6 h under argon followed by cooling, the ceramic boat with the content is quickly removed in ambient air from the tube flashed with argon and placed in the glove box (<1 min).

For characterization, in all cases a fast transfer of a sample to an analytical instrument is performed depending on the method’s specifics; for instance;

- For Fourier transform infrared spectroscopy (FT-IR) determinations, a sample is mixed with KBr in the glove box, and the mixture is placed in the tightly assembled tablet press, which is taken out and pressed in the air and, next, transferred to an infrared spectrometer for measurements (<2 min);
- For ultraviolet–visible spectroscopy (UV-Vis) determinations, in the glove box a sample holder is filled with a reference BaSO_4_ powder with a sample atop, and the setup is then removed and transferred in the air to a spectrometer (<1 min);
- For helium density determinations, in a glove box a weighed sample is placed in a 1 cm^3^ sample chamber that is transferred in the air for measurements under helium flow (<2 min);
- For powder X-ray diffraction (XRD), X-ray photoelectron spectroscopy (XPS), Raman spectroscopy, combined thermogravimetric analysis/differential thermal analysis–quadrupole mass spectrometry (TGA/DTA-QMS), and solid-state ^65^Cu/^119^Sn magic angle spinning nuclear magnetic resonance (^65^Cu/^119^Sn MAS NMR) determinations, the sample is prepared in a glove box under argon in a tightly closed vial and then promptly transferred to an instrument, and the measurement is immediately initiated.

### 2.2. Sample Labeling

The samples are identified by using the acronyms of the parent precursor system, i.e., either CE, MS, or CA (see above), and the stage of nanopowder thermal processing equivalent to the phase transition from the cubic to tetragonal polytype, i.e., raw powder (cubic prekesterite) and thermally treated powder (tetragonal kesterite).

### 2.3. Characterization Methods

Powder XRD determinations were performed on Empyrean PANalytical (Malvern, UK), Cu Kα source, 2Θ = 10–110°, and average crystallite sizes were calculated from Scherrer’s equation. An ultra-high-resolution analytical FIB-SEM Scios 2 scanning electron microscope (Thermo Fisher Scientific, Waltham, MA, USA) was used for particle morphology observations. FT-IR spectroscopy (Nicolet 380, Thermo Electron Corp., Waltham, MA, USA) was performed on KBr pellets containing about 1 mg of samples. Raman spectroscopy was conducted with a WITec Alpha 300M+ spectrometer (WITec, Ulm, Germany) equipped with a 488 nm diode laser. Typically, four accumulations of 30 s scans were collected at each point. Deconvolution of spectra was performed using a mixed Gaussian–Lorentzian fitting procedure. UV-Vis measurements were carried out with a PerkinElmer spectrophotometer Lambda 35 equipped with a 50 mm integrating sphere for powder samples (Waltham, MA, USA). Materials from the MS and CA systems were investigated by photoelectron spectroscopy XPS (Vacuum Systems Workshop Ltd., Crowborough, UK), using Mg anode with photon energy of 1253.6 eV, and referencing to C 1s peak at 284.8 eV. Solid-state MAS NMR spectra were acquired on the APOLLO console (Tecmag, Houston, TX, USA) at the magnetic field of 7.05 T with the Bruker HP-WB high-speed MAS probe (Billerica, MA, USA) equipped with the 4 mm zirconia rotor and KEL-F cap. The ^65^Cu NMR spectra were determined at 85.11 MHz with a spinning speed of 6 kHz. The frequency scale in ppm was referenced to the ^65^Cu resonance of CuCl. The ^119^Sn NMR spectra were measured at 111.68 MHz with a spinning speed of 6 kHz. The frequency scale in ppm was secondary-referenced to the central transition of SnS spectrum located at −299 ppm. For quantitative estimations of the ^65^Cu spins in a sample, a known amount of CuCl was added to a known amount of the tetragonal kesterite sample to enable calculations of the spins based on comparison of both signals’ relative intensities. The TDA/DTA-QMS measurements were performed using the thermal analyzer STA 449 F3 Jupiter Netzsch (Netzsch Gerätebau GmbH, Selb, Germany) in the temperature range from ambient to 1000 °C with a heating rate of 10 °C/min and a sample of ca. 30 mg. The thermogravimetric and thermal analyses were performed for each nanopowder under the neutral gas atmosphere of argon. The off-gas analysis was performed by the quadrupole mass spectrometer QMS 403C Aëolos that was coupled to the TGA/DTA analyzer by a heated quartz capillary. BET (Brunauer–Emmett–Teller)/BJH (Barrett–Joyner–Halenda) specific surface areas were determined from low-temperature nitrogen adsorption isotherms on Micromeritics Gemini 2380 (Norcross, GA, USA). Helium densities were obtained with a Micromeritics AccuPyc 1340 pycnometer (Norcross, GA, USA). The d_He_ values for the samples were rounded up to the nearest 0.001 g/cm^3^ and showed with a standard deviation value. The oxygen and hydrogen contents were directly determined with the ONH836 elemental analyzer (Leco Corporation, St. Joseph, MI, USA) using 0.01–0.02 g of a sample.

## 3. Results and Discussion

The three precursor systems processed along the anaerobic pathways of the mechanochemical synthesis afforded six nanopowders, i.e., three raw cubic prekesterites and three thermally annealed tetragonal kesterites. This constitutes a relatively large pool of nanopowders to follow the oxygen footprint from the initial precursor systems to the synthesis stages and the characterization of both polytypes. Such an approach was undertaken in order to avoid a somewhat limiting case-study syndrome when concentrating on one material by looking rather at a wide range of similarly made nanopowders to obtain a more reliable averaged-out picture of oxygen contents. The collected data were to be compared with the already published characteristics of the nanopowders made under the standard conditions.

**Figure 1:**
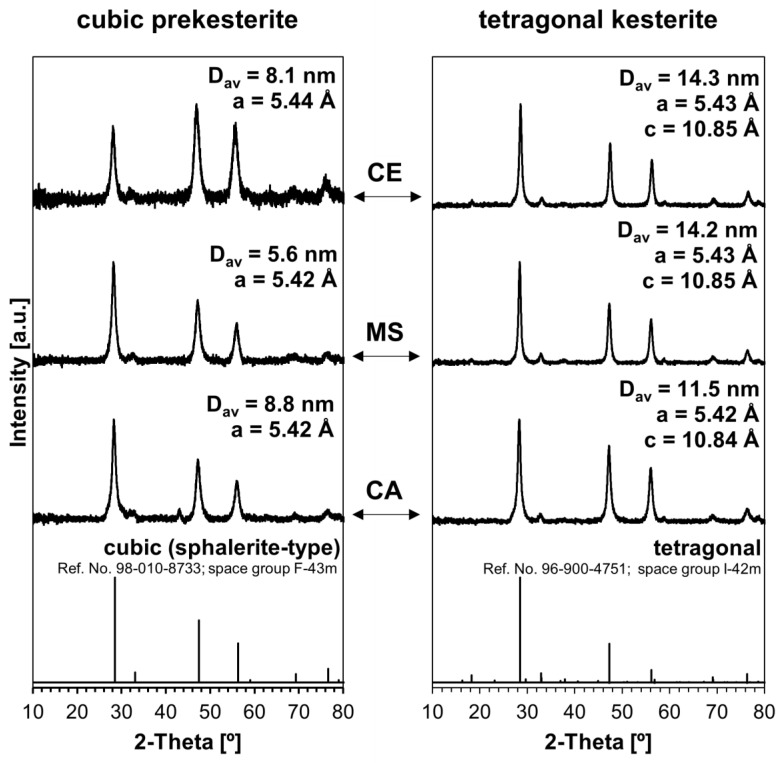
Powder XRD patterns for cubic prekesterite (**left column**) and tetragonal kesterite (**right column**) polytypes prepared via the anaerobic mechanochemical synthesis method from three different precursor systems: CE (constituent elements), MS (metal sulfides), and CA (copper alloys). Reference bar charts for cubic sphalerite ZnS (**left column**) and tetragonal kesterite (**right column**), which are copied from cards with specific reference numbers in any of the PDF (Powder Diffraction File) databases, as well as assigned space groups, are shown in the bottom row. Patterns include data of the average crystallite sizes *D_av_* and the cell parameters *a* and *c*.

The powder XRD patterns for all prepared materials are shown in Figure 1. As expected, each of the raw nanopowders is confirmed to be the cubic polytype (sphalerite-type cubic, space group F-43m), whereas the 500 °C treated nanopowders are the tetragonal variety (“pseudocubic” tetragonal kesterite, space group I-42m with random Cu/Zn position distribution). The kesterite phase transformation involves also changes in the degree of structural disorder, including stacking faults [22]. No other phases are found, which is not surprising for these highly pure products; furthermore, this can be related to the ca. 0.1–1.0 wt% XRD detection limits, which, additionally, are compounded by severe peak broadening for nanosized materials [23]. The crystallite sizes for the cubic prekesterites in the range of 5.6–8.8 nm are smaller than the sizes for the thermally treated tetragonal kesterites, the latter spanning 11.5–14.3 nm. The respective cell parameters are close for each of the polytypes while showing small variations among the precursor routes. In general, the spectra confirm the completion of the complex synthesis in all routes.

**Figure 2:**
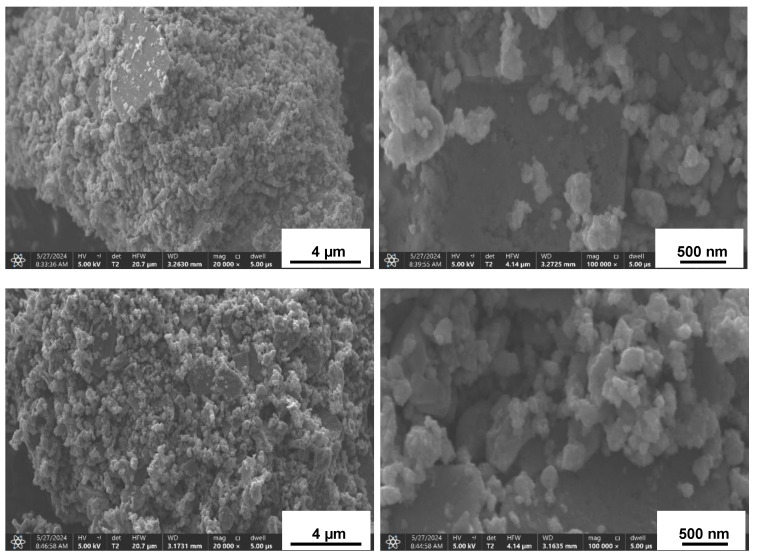
Typical SEM images of all nanopowders exemplified with different magnifications for CE precursor system products: **upper row**—prekesterite, **bottom row**—kesterite.

The SEM examination of the nanopowders confirms the overall similarity of the prekesterites and kesterites, and an example of the typical particle morphology is shown for the CE system in Figure 2. The images present highly agglomerated and aggregated particles down to the nanosized regime, as already reported by us for the mechanochemical synthesis method on other occasions [16,17,19,21]. In the highest magnifications (right column), particles with sizes below 100 nm are clearly discernible. It is instructive to note that the particle sizes are not directly comparable with the XRD-derived average crystallite sizes, since most of the particles are just agglomerates of much smaller crystallites.

**Figure 3:**
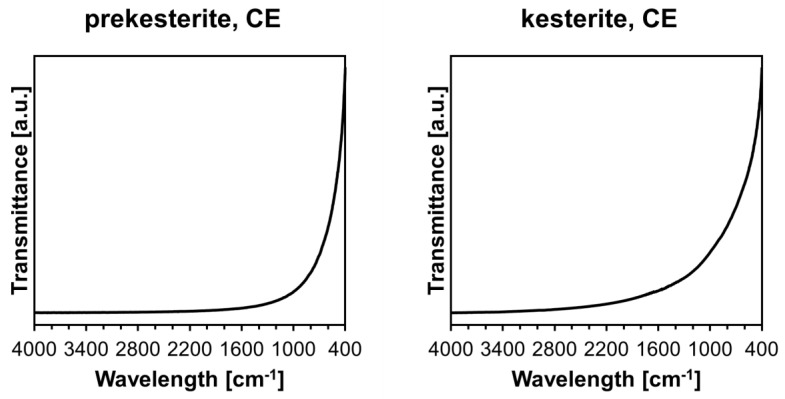
FT-IR spectra of prekesterite and kesterite nanopowders prepared from CE precursor system. Note that the curvature of the baseline is due to nonspecific light absorption in KBr pellets.

The infrared FT-IR spectra are identical for all prekesterites and kesterites from the three precursor systems. In this regard, the kesterite has no specific bands in the mid-infrared range, and this is reflected in the featureless spectra shown as an example for the CE system in Figure 3. The baseline curvature in the spectra is due to secondary non-specific interactions of the probing laser beam, mainly scattering and background measurements, with solid particles in the KBr pellet. It is worth mentioning that the FT-IR spectra even for the “freshly made” nanopowders prepared via the standard procedure were previously found to have weak bands at ca. 1620, 1100, and 620 cm^−1^ for the hydrated Cu/Zn sulfates—the products of kesterite oxidation in ambient air [16,17]. The current data favorably show no signs of such oxidation, at least at the method’s detection limits.

**Figure 4:**
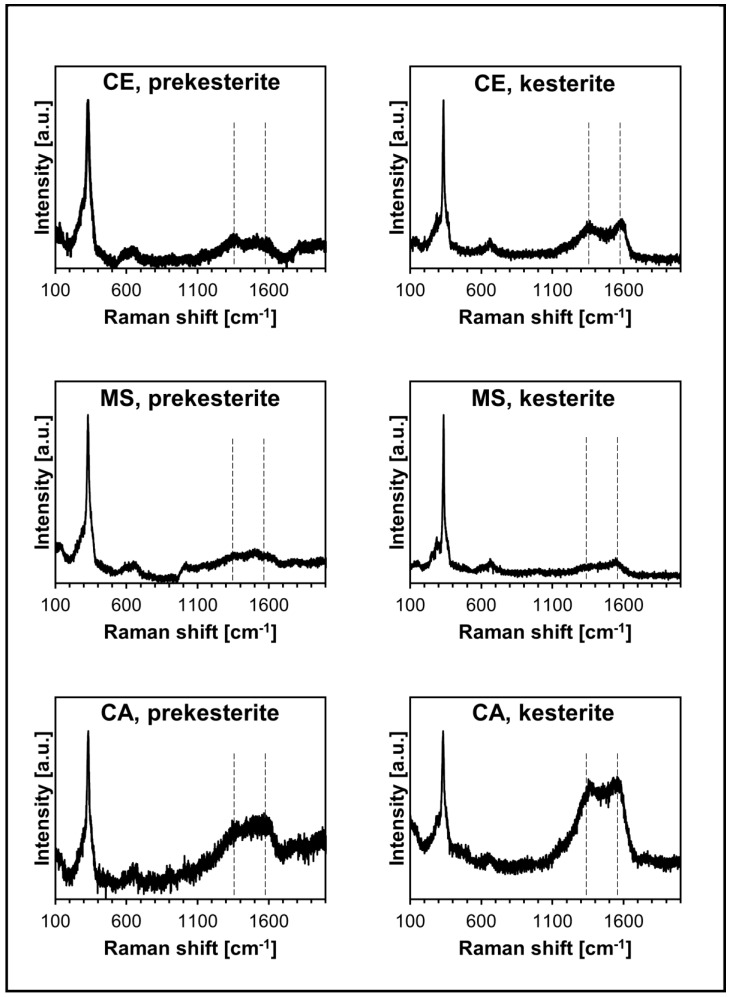
Micro-Raman spectra for prekesterites (**left**) and kesterites (**right**) from CE, MS, and CA precursor systems. Vertical dashed lines are in positions of residual carbon and are guides for the eye, only.

The micro-Raman spectra of all nanopowders are shown in Figure 4. The spectra are typical for the kesterite polytypes, as previously reported by us [16,17,19] and others [24].

The signature feature includes three closely-spaced bands at ca. 290–300 (weak), 328–330 (strong), and 348–350 cm^−1^ (weak, broad), which are broader and severely superimposed for the prekesterite and sharper and often separated for the kesterite. Also, a weak, broad peak is observed at ca. 650–660 cm^−1^, which we tentatively assign to the overtone of the most intense peak at 328–330 cm^−1^. The resemblance of the Raman spectra for the cubic prekesterite and tetragonal kesterite suggests their similar chemical bonding and lattice phonon characteristics. The typical feature of the spectra observed here is also a pair of two broad and superimposed bands at ca. 1370 and 1570 cm^−1^ that we assign to residual carbon. Its likely source is the remnants of xylene that, apparently, are not efficiently removed from the raw powder even upon application of vacuum (see Section 2) and decompose during the thermal treatment at 500 °C. Interestingly, these bands, while broader, are also seen in the raw prekesterites that were not thermally treated. By comparison with the Raman spectra for different xylene isomers, the bands can hardly be assigned to them [25]. However, the high-energy ball milling applied in the synthesis may involve a local increase in particle temperature up to several hundred degrees that could effect some xylene breakdown and nanocarbon formation. This, then, could be a non-volatile source of carbon in the resulting thermally treated kesterite. Interestingly, it appears that the nanopowders from different precursor systems contain varying relative amounts of carbon, and the least carbon-contaminated products are from the MS precursor system. And finally, there are no signs of the bands for the SO_4_^2−^ sulfate groups at 440–470 and 1000–1020 cm^−1^ that were seen previously for such nanopowders after their prolonged exposure to air and that were assigned to the presence of the hydrated copper and zinc sulfates.

**Figure 5:**
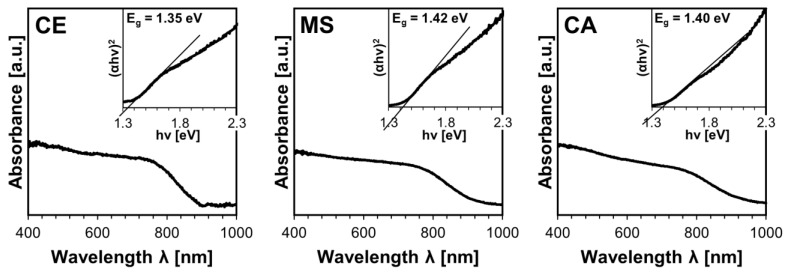
UV-Vis spectra for thermally annealed tetragonal kesterite nanopowders from CE, MS, and CA precursor systems. In inserts, Tauc (αhν)2 vs. hν [energy] plots (α approximated by Kubelka–Munk transformation), and the energy band gaps E_g_ derived from them, are shown.

The property of the kesterite semiconductor crucial to photovoltaic applications is its energy band gap, E_g_. At this point, it is recalled that the nanopowders of the raw cubic prekesterite show defunct semiconductor properties, and no band gap can be derived from the unspecific UV-Vis data. For the annealed tetragonal kesterite nanopowders, the band gap is calculated from UV-Vis measurements upon application of the Kubelka–Munk transformation via Tauc’s function by plotting a tangent curve and recording its intersection with the energy axis, as shown in Figure 5. The E_g_ values in the range 1.35–1.42 eV in this study are typical for the kesterite and can be compared with our previously accumulated data for such nanopowders [17,19,20,21], i.e., for the CE system—1.35 eV vs. 1.40 eV, 1.41–1.48, and 1.40 eV; for the MS system—1.42 eV vs. 1.38 eV and 1.45 eV; for the CA system—1.40 eV vs. 1.35 eV and 1.55 eV. It appears that small variations in the E_g_ values arise from slight differences in the synthesis parameters such as sulfur S excess, rotation speed and milling time, mass of the charge, annealing temperature, etc. Also, the extensive materials oxidation was shown to result in shifting the band gap to lower values by 0.10–0.20 eV for the kesterites from all three precursor systems. Overall, we consider the relatively small range of E_g_s measured in this study as reflecting the consistency of the anaerobic synthesis approach.

**Figure 6:**
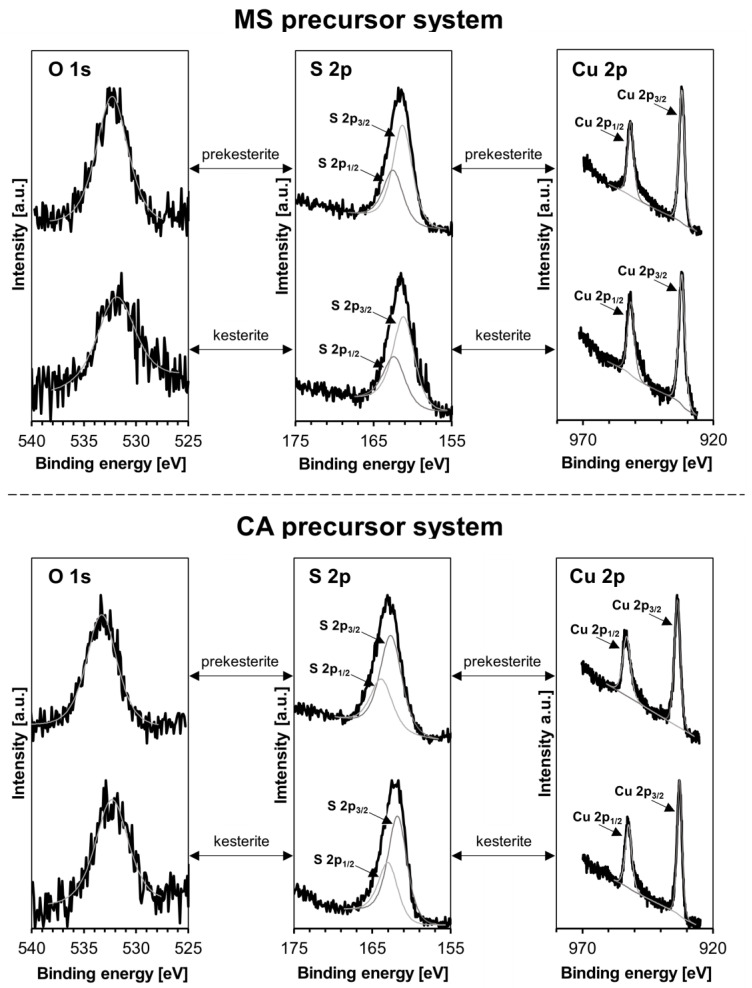
Oxygen O 1s, sulfur S 2p, and copper Cu 2p XPS spectra for nanopowders from MS and CA precursor systems. For each system, XPS signals for prekesterite are shown in the upper row and for kesterite in the bottom row.

X-ray photoelectron spectroscopy (XPS) is a surface-sensitive technique that probes the chemical environment down to ca. 10 nm in depth. In our earlier report on the magnetic properties of kesterite, we described the XPS data for the nanopowders from the MS and CE systems prepared in the standard way [20]. Specifically, the Cu 2p_1/2_ and Cu 2p_3/2_ peaks confirmed the presence of the Cu^1+^ centers, as expected for kesterite, whereas no clear signs of Cu^2+^ were seen to imply extensive surface oxidation. However, the S 2p_1/2_ and S 2p_3/2_ peaks, in addition to the strong signals at 161.5–162.2 eV typical for the sulfide S^2−^ ions in kesterite, showed also the doublet in the range 168.8–169.2 eV consistent with various inorganic sulfates (SO_4_^2−^ groups). These peaks were observed for both the prekesterite and kesterite and provided strong arguments for the adventitious kesterite oxidation toward metal sulfates on exposure to ambient air. In this study, the XPS measurements are carried out for the MS and CA precursor systems, and the oxygen O 1s, sulfur S 2p_1/2_/S 2p_3/2_, and copper Cu 2p_1/2_/Cu 2p_3/2_ peaks are shown in Figure 6; the positions of all peaks are included in Table 1.

The data fully confirm the completeness of the synthesis reactions and the formation of the kesterite nanopowders. In this regard, the standard way to report the S 2p_1/2_/2p_3/2_, Cu 2p_1/2_/2p_3/2_, Zn 2p_1/2_/2p_3/2_, and Sn 3d_3/2_/3d_5/2_ doublets is by reporting a position of the higher intensity/lower energy peak (all shown in bold in Table 1). These peaks confirm the S^2−^ sulfide character of sulfur and Cu^1+^, Zn^2+^, and Sn^4+^ oxidation states for the metals as established for kesterite [26]. Of particular significance is the lack of sulfur peaks in the sulfate range. Also, there are no satellites observed in the Cu 2p spectrum, and this excludes detectable surface Cu^2+^ ions that could form via kesterite oxidation. Some oxygen is yet detected, and the O 1s peak in the range 532.5–533.0 eV is assigned to surface adsorbed water. Apparently, despite the time-limited and relatively short exposure to air, mainly during sample preparation and manipulations in the XPS measurements, some water is yet adsorbed. It is also noticed that there are no essential differences in peak positions between the cubic prekesterite and tetragonal kesterite from both precursor systems, and this is consistent with the basically very similar chemical environment in both polytypes.

**Figure 7:**
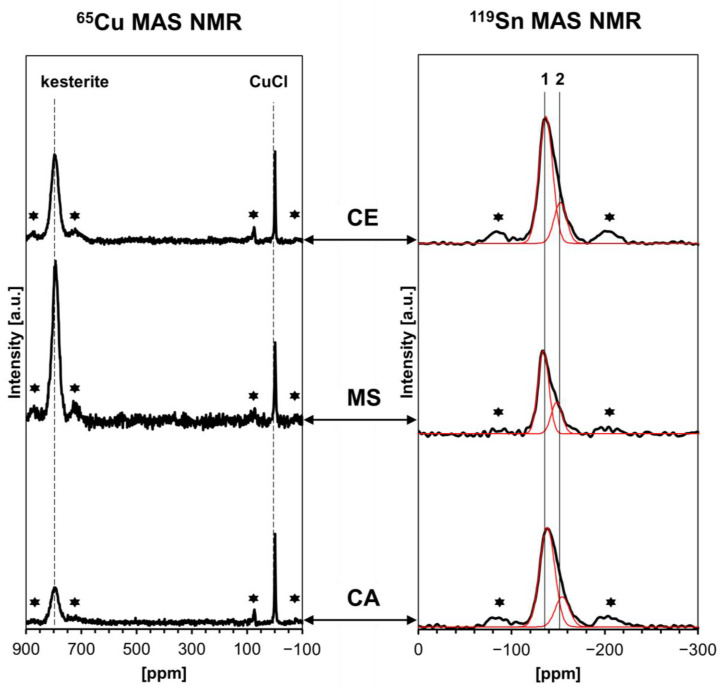
^65^Cu (**left**) and ^119^Sn (**right**) MAS NMR spectra for annealed kesterite nanopowders from CE, MS, and CA precursor systems. Dashed vertical lines in left column show ^65^Cu peak positions for kesterite (broad peak) and CuCl (sharp peak, 0 ppm). Solid vertical lines in the right column show approx. positions of two deconvoluted ^119^Sn peaks shown in red—labeled 1 and 2. The lines are guides for the eye only. Asterisks (*) indicate spinning sidebands.

The solid-state ^65^Cu/^119^Sn NMR spectroscopy has shown to be a versatile technique in kesterite characterization and has been applied in studies on a few occasions [27,28,29], including our contribution [16,17,19,20,21]. Interestingly, we found that the raw cubic prekesterite nanopowders do not produce ^65^Cu/^119^Sn MAS NMR signals at all, which we attributed to intrinsic d^0^ magnetism in the defected freshly made particles prepared via the high-energy ball milling. The existence of magnetism without d-orbital participation, for instance, such as the ferromagnetism observed in “non-magnetic” oxides or nitrides, is proposed to emerge due to the polarization induced by p-orbitals [30]. On the other hand, after thermal annealing, the tetragonal kesterite nanopowders show the expected ^65^Cu/^119^Sn NMR resonances, with, however, somewhat adventitiously scattered signal intensities. For instance, we observed a pronounced difference in the mass-normalized peak intensities between the kesterite samples from the MS system that were annealed at 500 and 550 °C. Further, a calibration of these peak intensities vs. the CuCl reference proved a mere 12 and 24% of all nuclei to be active in the NMR experiments, respectively. This had to be confronted with the still measurable, though decreased, magnetization in the annealed kesterite samples compared with the parent prekesterites. Although a precise interrelation between magnetization and activity of the copper and/or tin nuclei in the NMR experiment is not clear at this point, the knowledge about the share of the actively resonating nuclei may provide useful clues related to the completeness of element ordering in the lattice. Figure 7 presents the ^65^Cu (left column)/^119^Sn (right column) MAS NMR spectra for the kesterite nanopowders from all three precursor systems. The samples include a known quantity of CuCl to be used for estimating via calibration the proportion of the NMR-active copper centers in kesterite. The asymmetry of the ^119^Sn peaks suggests the presence of two superimposed peaks, as supported by deconvolution, assuming the isotropic tin nuclei with spin ½. 

The numerical data extracted from the spectra are shown in Table 2. The ^65^Cu chemical shifts are in the range 794.7–797.0 ppm (column 1) to be compared with our previously determined values of 792–799 ppm. The ^119^Sn peaks show, this time, clear asymmetry in all cases and after deconvolution, provide for dominant peak 1 shifts of −133.2–137.5 ppm and for peak 2 shifts of −147.4–154.1 ppm (columns 5–7). In this regard, in our previous study on the CA system processed in the standard way, one of the kesterite products showed also such peak asymmetry although with slightly different chemical shifts upon deconvolution, i.e., −128.1 and −139.0 ppm, respectively, while with very similar proportions of 78 and 22%. It is to be recalled that the ^119^Sn peak asymmetry was also observed in other studies on kesterite [27,31]. The reason for the existence of two subsets of tin nuclei may be the symmetry differentiated Cu nuclei in the 2a and 2c sites discriminated by distinct S-Cu-S angles, which may propagate differences through the S layer to the neighboring Cu/Sn layer and differentiate the Sn atoms there [32]. It is remarkable, though, that the relative intensities of the two deconvoluted peaks are quite well reproducible in all cases, indicating the approximate ratio 3:1 for the two different ^119^Sn chemical shift environments.

In the calibration of the Cu signals, column 4 in Table 2 summarizes the calculations by showing the ratio of the copper signal intensity in the added CuCl to the copper signal intensity in kesterite expressed as for 1 mg of CuCl per 1 mg of kesterite. This ratio is equal to the molar ratio of the NMR active Cu atoms in these two compounds. In this regard, if one assumes the stoichiometric kesterite Cu_2_ZnSnS_4_ (MW of 439.47 g/mol) and CuCl (MW of 99.00 g/mol) with all NMR active Cu nuclei, the theoretical and, at the same time, minimal value of the ratio is calculated as 2.21. It appears that the kesterite from the CE system with the measured ratio 2.24 is very close to this reference value, whereas the ratios for the MS system of 4.74 and CA system of 4.00 suggest a range with close to half of the kesterite molecules to be actively contributing to the copper signal intensity. These are not surprisingly low values at all, since in our earlier NMR studies on the systems processed in the standard way, some 22–23% of resonating copper nuclei were calculated for the CA system and 12–24% for the MS system. Apparently, the anaerobic synthesis routes result in kesterite nanopowders with significantly lower proportions of resonance-disabled copper centers in the kesterite crystal lattice, and it is tempting to link this with the accompanying restricted oxidation. Although the exact nature of the factors behind some of the theoretically Cu d^1^ atoms in kesterite not resonating in the NMR experiment is unclear, the ^65^Cu/^119^Sn MAS NMR with CuCl calibration certainly enables us to appraise the progress of oxidation, completeness of reactions, and extent of disorder of the kesterite lattice. And it helps to compare the various precursor system routes from these points of view.

Thermogravimetry coupled with mass spectrometry TGA/DTA-QMS were previously used by us, mainly, to look into various gas species being evolved upon temperature rise from the kesterite polytypes prepared by the standard routes from the CE and CA precursor systems [18]. A range of volatile compounds containing oxygen, such as water vapor H_2_O, carbon dioxide CO_2_, sulfur dioxide SO_2_, and sulfur trioxide SO_3_, was found in all cases. The presence of H_2_O, SO_2_, and SO_3_ could be linked to some oxidized kesterite nanopowders with the formation of hydrated Cu/Zn sulfates that were then decomposing under the rising temperature conditions of the experiment. CO_2_ was proposed to be an artifact of using liquid xylene (“wet” milling, see Section 2) that was not efficiently evaporated in the final mechanochemical synthesis step. The xylene/hydrocarbon remnants or nanocarbons reacted further with SO_3_ and/or SO_2_ toward the formation of CO_2_ but also, possibly, of carbonyl sulfide COS and carbon disulfide CS_2,_ in a few distinct temperature ranges spanning ca. 300–900 °C (prekesterites) or 300–700 °C (kesterites).

**Figure 8:**
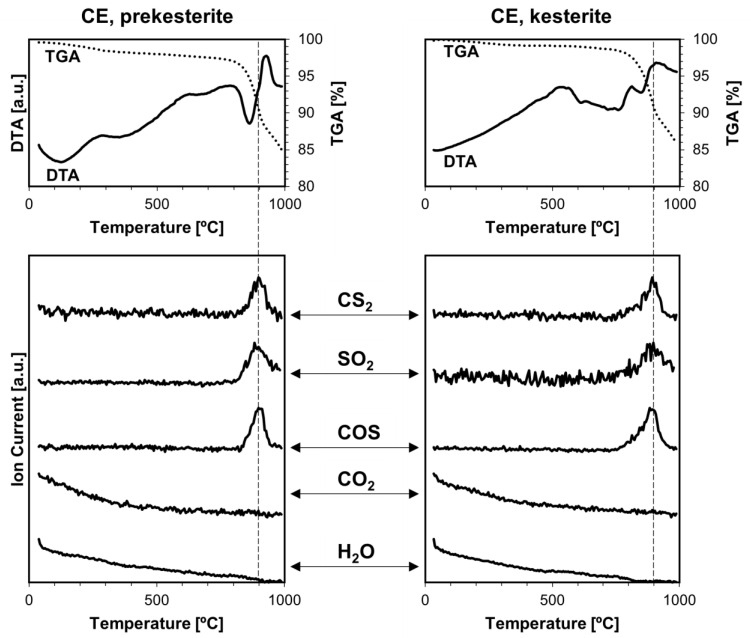
TGA/DTA-QMS determinations for prekesterite (**left**) and kesterite (**right**) nanopowders from CE precursor system. The top row shows thermogravimetric (TGA—dotted line) and thermal (DTA—solid line) changes, whereas the rows below show gas evolution curves for m/e corresponding to, from top to bottom, CS_2_, SO_2_, COS, CO_2_, and H_2_O. Vertical dashed lines indicate peak positions of evolving compounds and are guides for the eye, only.

**Figure 9:**
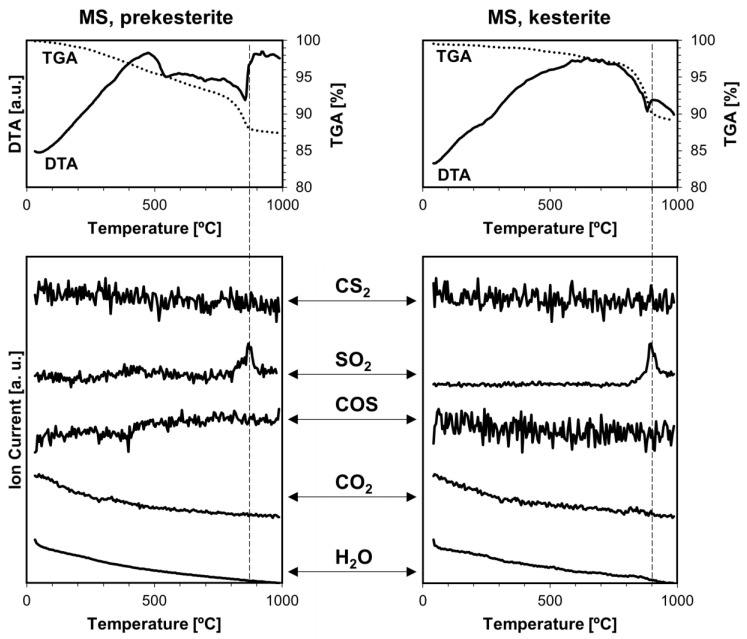
TGA/DTA-QMS determinations for prekesterite (**left**) and kesterite (**right**) nanopowders from the MS precursor system. The top row shows thermogravimetric (TGA—dotted line) and thermal (DTA—solid line) changes, whereas the rows below show gas evolution curves for m/e corresponding, from top to bottom, to CS_2_, SO_2_, COS, CO_2_, and H_2_O. Vertical dashed lines indicate peak positions of evolving compounds and are guides for the eye, only.

**Figure 10:**
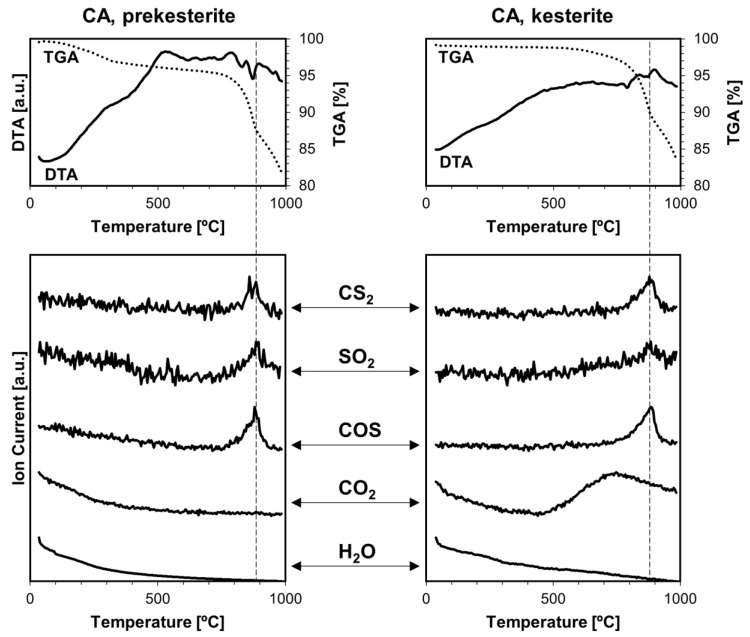
TGA/DTA-QMS determinations for prekesterite (**left**) and kesterite (**right**) nanopowders from the CA precursor system. The top row shows thermogravimetric (TGA—dotted line) and thermal (DTA—solid line) changes, whereas the rows below show gas evolution curves for m/e corresponding, from top to bottom, to CS_2_, SO_2_, COS, CO_2_, and H_2_O. Vertical dashed lines indicate peak positions of evolving compounds and are guides for the eye, only.

Figure 8, Figure 9 and Figure 10 show the current results of the TGA/DTA-QMS experiments under argon for the nanopowders of both kesterite polytypes and all three precursor systems. The TGA/DTA curves for the prekesterite (left) and kesterite (right) are shown in the top row of each figure. First, the curves confirm the beginning of noticeable decomposition under the experimental conditions with mass losses of both polytypes starting at ca. 700–800 °C, at which point the losses visibly accelerate. The decomposition stage is associated with one or more DTA endothermic effects. Second, before that stage, the mass loss for each prekesterite is larger compared with the related kesterite, confirming the better thermal stability of the latter. And third, the TGA curves for the CE and CA systems are quite similar and differ from the one for the MS system, the latter showing the relatively lowest mass losses and clearly slowing the decomposition rate before reaching the final 1000 °C. This can be possibly traced to the apparently lowest carbon contents in the polytypes from the MS system, as supported by the Raman spectra (Figure 4) and, consequently, the minimal if any reactions toward volatile CS_2_ and COS. The kesterite polytypes from the CE and CA systems show also similar mass spectra that support the evolution of the volatile gases, whereas those from the MS system show only the evolution of SO_2_. All gases are detected in the kesterite thermal decomposition range of 800–900 °C. It is worth recalling that the similar analytical data for the kesterite polytypes prepared via the standard synthesis showed also, in addition to the decomposition high temperature range above ca. 800 C, evolution of SO_2_ and SO_3_ at a lower temperature range below 500–600 °C, which was linked to the decomposition of the metal sulfates formed upon kesterite adventitious oxidation in air. Also, the CO_2_ evolution was quite extensive and complex then, compared with no evidence for its evolution at all, now. In order to clarify the issue of the detection of CS_2_ and COS in this study, we admit that these compounds (appropriate m/e values) were not targeted previously due to a supposed low probability of their occurrence, but they were analyzed now with success.

The density and porosity of the nanopowders have a direct impact on their stability in air by linking these properties with particle surface characteristics and the propensity to adsorb/chemisorb various gases. The helium densities and BET/BJH-specific surface areas for all nanopowders are shown in Table 3. The magnitudes of the helium density for both polytypes are in the range of 3.959–4.443 g/cm^3^ and can be compared with our previously determined values of 3.6–4.3 g/cm^3^ for the nanopowders made in a standard way and with the available reference value for tetragonal kesterite of 4.56 g/cm^3^ [33]. Generally, the current densities are higher overall than those from the previous study, supporting now a more pristine particle surface morphology. In this regard, the air-induced oxidation of the nanopowders was shown to result in a relative density decrease. In the MS and CA systems, the densities of the annealed kesterites are a bit higher than those of the respective raw prekesterites, which is consistent with a more defected structure of the latter. The inverse order for the CE system could be explained by a specific increase in helium-inaccessible closed pores in the annealed kesterite compared with the raw prekesterite. The BET-specific surface areas (considered total areas) are also higher now, by almost a factor of 2, than were found earlier for the standard synthesis, with the latter showing ca. 22 m^2^/g as the highest area, to be compared now with the highest at 44 m^2^/g. The values of the BJH-specific surface areas (related to mesopore areas) are close to the respective BET numbers, which is consistent with the prevailing mesoporosity of all nanopowders as was also the case in the standard synthesis. Interestingly, the magnitudes of the BET/BJH surface areas (m^2^/g) are very similar for both polytypes in all systems, with slightly lower values for prekesterite vs. kesterite in the CE system, i.e., 28/33 vs. 33/73; inversely higher values for prekesterite vs. kesterite in the MS system, i.e., 44/62 vs. 36/42; and essentially very similar values for both polytypes in the CA system, i.e., 40/51 vs. 41/53, respectively. This is compliant with only small, if any, morphology changes occurring upon the thermal treatment and accompanying phase transition from the cubic to tetragonal polytype. In concluding these aspects, the density and specific surface area parameters of the anaerobic nanopowders describe more realistically these properties and can be convenient reference points for plausible oxidation-related changes.

Finally, all the characterization data discussed above face the suitable ramifications by relating to the directly determined oxygen and hydrogen contents shown for the nanopowders in Table 4 and for the metal and metal sulfide precursors in Table 5. The data were acquired for the freshly made nanopowders, followed by their exposure to ambient air for 24 h, 48 h, and 7 days and the O- and H-analyses performed after each of these periods of time. Such times of air exposure were intended to fill in the short-term gap in the previous study, when the analyses were carried out after much longer 1, 3, and 6 months of exposure [17].

First, the O contents are visibly lower now than in the previous study for both the prekesterites and kesterites. The current ranges for the freshly made prekesterites of 0.44–1.44 wt% and kesterites of 0.19–0.65 wt% can be compared with the respective ranges of 4.32–5.86 wt% and 0.63–1.60 wt% earlier. The O contents are now clearly lower by up to one order of magnitude, especially for the prekesterites. Also, both studies confirm a trend of lower O contents for the respective tetragonal kesterite in all three related polytype pairs, which is consistent with its relatively lower reactivity toward oxidation. Second, there are differences among the nanopowders from different precursor systems in the progress of oxidation up to one week of exposure to air. The prekesterites show the O content increases by some 4 to 6 times with a notable exception of the prekesterite from the most robust CA system which shows less than a two time increase, while for all kesterites the increases are close to two times. These exposure time/O content ranges can be referred to the much longer exposure times in the standard synthesis case, when after 1 month, already more than 20 wt% O contents were found in all precursor systems. Third, the O contents are larger in the pool of metal sulfide precursors than in the pure metal precursors, and, especially, the commercial zinc sulfide ZnS has a relatively high O content of 2.33 wt%. The source of this oxygen content is unknown; it could be, for instance, associated with the high-temperature processing, upon which the oxygen is likely Zn-bound. In such a case, the reaction environment of the mechanochemical synthesis carried out with some excess of sulfur can remedy the problem by reducing the initial oxygen content impact on the final kesterite oxygen footprint. Undoubtedly, the exact bonding nature of small oxygen contents, including some oxygen for sulfur replacement in the kesterite lattice, is a separate subject requiring specific analytical/characterization techniques.

## 4. Conclusions

The nanopowders of the cubic sphalerite-type and tetragonal polytypes of the kesterite Cu_2_ZnSnS_4_ semiconductor made via the mechanochemically assisted synthesis method are found to be prone to oxidation in ambient air, which is detected in most cases already after 24 h exposure times. The raw cubic prekesterite is much more reactive than the thermally annealed tetragonal kesterite, while the rate of deteriorating reactions depends on a specific precursor route. By referring to the standard mechanochemical synthesis where the pronounced long-term oxidation was confirmed, now, a modified preparation procedure is demonstrated to significantly reduce the adventitious oxygen contents in the freshly made kesterite products from all precursor routes. The modifications include the application of anaerobic steps whenever possible and the rational minimization of nanopowder contact times with air whenever such contacts are unavoidable. This requires the use of an inert gas glove box and a vacuum–inert gas Schlenk technique for material manipulations, the latter mainly for removal of the volatile hydrocarbon xylene used in the “wet” high-energy ball milling step. The applied modifications result in freshly made nanopowders with O contents mostly below 1 wt% to be compared with contents of up to ca. 6 wt% in the standard synthesis method. Significantly, the O contents in the thermally annealed kesterite nanopowders are now in the low range of 0.19–0.65 wt%, and they appear to be oxidized in air at much slower rates than the related raw cubic prekesterites. Finally, the kesterite polytype nanopowders, and especially, the raw cubic polytype, should be considered to be oxidation reactive in ambient air and treated as such.

## Figures and Tables

**Table 1:** Energies of XPS peaks for prekesterite and kesterite nanopowders prepared mechanochemically from MS and CA precursor systems. Note that higher intensity/lower energy peaks of doublets are shown in bold.

	**Peak Positions [eV]**				
	**O 1s**	S 2p_1/2_/**S 2p_3/2_**	Cu 2p_1/2_/**Cu 2p_3/2_**	Zn 2p_1/2_/**Zn 2p_3/2_**	Sn 3d_3/2_/**Sn 3d_5/2_**
MS, prekesterite	**532.8**	163.0/**161.8**	952.3/**932.7**	1045.2/**1022.3**	495.3/**486.6**
MS, kesterite	**532.7**	163.1/**161.9**	952.1/**932.6**	1045.1/**1022.6**	496.1/**486.7**
CA, prekesterite	**533.0**	163.3/**162.1**	952.5/**933.1**	1045.9/**1022.6**	495.7/**486.8**
CA, kesterite	**532.5**	163.2/**162.0**	952.5/**933.0**	1045.7/**1022.6**	496.1/**486.7**

**Table 2:** Chemical shifts *δ* and relative intensities of resonances in ^65^Cu/^119^Sn MAS NMR spectra shown in Figure 7 for kesterite nanopowders with known amounts of added CuCl.

	^65^Cu MAS NMR				^119^Sn MAS NMR		
	kesterite δ [ppm]	CuCl δ [ppm]	Peaks Relative Intensity, kesterite/CuCl [%/%]	Intensities Ratio per 1 mg CuCl/1 mg kesterite	Peak 1 δ [ppm]	Peak 2 δ [ppm]	Peaks Relative Intensity, 1/2 [%/%]
	1	2	3	4	5	6	7
CE	796.6	0	88/12	2.24	−135.9	−152.0	76/24
MS	794.7	0	91/9	4.74	−133.2	−147.4	72/28
CA	797.0	0	74/26	4.00	−137.5	−154.1	79/21

**Table 3:** Helium density (standard deviation S.D.) and BET/BJH-specific surface area data for anaerobic nanopowders from three precursor systems. BET (Brunauer–Emmett–Teller) values correspond to total surface areas and BJH (Barrett–Joyner–Halenda) values to mesopore areas.

CE System		MS System		CA System	
prekesterite	kesterite	prekesterite	kesterite	prekesterite	kesterite
Helium density d_He_ (S.D.) [g/cm^3^]					
4.360 (0.005)	4.155 (0.006)	4.248 (0.009)	4.443 (0.004)	3.959 (0.008)	4.351 (0.007)
BET/BJH specific surface area [m^2^/g]					
28/33	33/73	44/62	36/42	40/51	41/53

**Table 4:** Directly determined oxygen and hydrogen contents in anaerobic nanopowders of both kesterite polytypes prepared from CE, MS, and CA precursor systems. Determinations were made for freshly made nanopowders and for nanopowders exposed to ambient air for 24 h, 48 h, and 7 days.

	CE System		MS System		CA System	
	prekesterite	kesterite	prekesterite	kesterite	prekesterite	kesterite
	oxygen content [wt%]					
freshly made	0.44	0.19	0.97	0.65	1.44	0.42
24 h	0.93	0.25	1.38	0.67	1.37	0.29
48 h	1.44	0.27	2.00	0.69	1.39	0.41
7 days	2.84	0.38	3.91	1.45	1.81	0.75
	hydrogen content [wt%]					
freshly made	0.27	0.15	0.22	0.09	0.35	0.05
24 h	0.28	0.16	0.25	0.07	0.35	0.04
48 h	0.32	0.15	0.27	0.08	0.35	0.07
7 days	0.37	0.15	0.34	0.12	0.35	0.16

**Table 5:** Directly determined oxygen and hydrogen contents in freshly acquired powders of the metal (copper Cu, zinc Zn, tin Sn) and metal sulfide (copper sulfide Cu_2_S, zinc sulfide ZnS, tin sulfide SnS) precursors.

	Cu	Zn	Sn	Cu_2_S	ZnS	SnS
oxygen content [wt%]	0.24	0.10	0.10	1.32	2.33	0.62
hydrogen content [wt%]	0.005	0.003	0.002	0.03	0.23	0.02

## Data Availability

The original contributions presented in this study are included in the article. Further inquiries can be directed to the corresponding author.
